# World Rabies Day – a decade of raising awareness

**DOI:** 10.1186/s40794-016-0035-8

**Published:** 2016-09-28

**Authors:** Deepashree Balaram, Louise H. Taylor, Kim A.S. Doyle, Elizabeth Davidson, Louis H. Nel

**Affiliations:** 1 Global Alliance for Rabies Control, 529 Humboldt St., Suite 1, Manhattan, KS 66502 USA; 2 Boyd Orr Centre for Population and Ecosystem Health, University of Glasgow, Glasgow, UK; 3 Department of Microbiology and Plant Pathology, Faculty of Natural and Agricultural Sciences, University of Pretoria, Pretoria, 0001 South Africa

**Keywords:** Rabies, Canine rabies, World Rabies Day, Rabies awareness, Rabies prevention, Canine rabies elimination

## Abstract

World Rabies Day was set up in 2007 to raise global awareness about rabies, to provide information on how to prevent the disease in at-risk communities and support advocacy for increased efforts in rabies control. It is held annually on September 28th, with events, media outreach and other initiatives carried out by individuals, professionals, organisations and governments from the local to the international level.

The Global Alliance for Rabies Control coordinates World Rabies Day, amplifying the campaign’s reach through the provision of a central event platform and resources to support events across the world, the promotion of messages through key rabies stakeholders, and the implementation of specific activities to highlight particular issues. Over the last decade, more than 1,700 registered events have been held across the world and shared with others in the global rabies community. Events in canine rabies endemic countries, particularly in Africa and Asia, have increased over time. Beyond the individual events, World Rabies Day has gained the support of governments and international agencies that recognise its value in supporting existing rabies control initiatives and advocating for improvements.

As the rabies landscape has changed, World Rabies Day remains a general day of awareness but has also become an integral part of national, regional and global rabies elimination strategies. The global adoption of 2030 as the goal for the elimination of rabies as a public health threat has led to even greater opportunities for World Rabies Day to make a sustainable impact on rabies, by bringing the attention of policy makers and donors to the ongoing situation and elimination efforts in rabies-endemic countries.

## Background

### The global rabies situation

Rabies is a zoonotic viral disease that is transmitted through the saliva and nervous tissue of an infected animal. It is listed as one of 18 Neglected Tropical Diseases by the World Health Organization [1]. Rabies is almost always fatal—it has one of the highest case fatality rates of any disease [2]. However, rabies is also 100 % preventable, through human post-exposure prophylaxis (PEP), improved educational awareness to prevent exposure, and mass vaccination of dog populations.

More than 99 % of all rabies cases in humans are transmitted from dogs [2]. Despite proven methods of elimination, an estimated 59,000(95 % CIs:25–159,000) people die each year due to dog-transmitted rabies, with over 95 % of these deaths occurring in Africa and Asia [3]. The fear of rabies is also responsible for many animal deaths each year – not only do they suffer from the disease’s horrific clinical symptoms, but dogs in particular are killed in large numbers in futile and often inhumane attempts to control the spread of the virus.

Where political will and funding exist, canine rabies can and has been eliminated [4, 5], but the proven methods based primarily on dog vaccination have not been applied everywhere. A decade ago, efforts to end rabies were not progressing across Africa and Asia. Thousands of people were still dying in neglected communities, there was a lack of collaboration among international stakeholders, experts and sectors and there was little evidence of successful rabies control programmes in resource poor areas.

The Global Alliance for Rabies Control (GARC) was established to address these issues, and over the last ten years, GARC has worked with international stakeholders, including the United Nations Food and Agriculture Organization (FAO), World Organisation for Animal Health (OIE), World Health Organization (WHO) and many other partners to raise awareness about rabies, encourage collaboration and build the evidence base needed to increase political commitment and funding to end rabies in every country. More recently, large-scale intersectoral rabies projects in Asia and Africa have demonstrated that existing effective vaccines can be applied in diverse settings and reach enough dogs to break the cycle of transmission [6]. The feasibility of canine rabies elimination through mass dog vaccination is now widely accepted [2].

However, despite the evidence of feasibility, global elimination still remains a large challenge. Adequate funding for rabies elimination, both internationally and at a national level, as well as prioritisation of the disease by governments of rabies-endemic countries, are now the primary elements needed to end the disease. A major step forward occurred in December 2015, when member countries at a global meeting organised by the WHO, OIE, FAO and GARC, agreed on a global strategic framework for the elimination of the public health threat of rabies by 2030 [7].

### The need for World Rabies Day

World Rabies Day was the first initiative of GARC to unite the rabies community to speak together and raise awareness of the issue. Its development was supported by GARC’s Partners for Rabies Prevention, during an analysis of barriers to more successful rabies control efforts and possible solutions, who recognised its potential to have an impact [8] (Table 1).

**Table 1:** Gaps identified in rabies prevention where World Rabies Day can contribute

Gap identified	Relevant target audiences
Human rabies prevention: Lack of awareness about appropriate rabies prevention strategies	Public health practitioners and community members
Animal rabies control: Poor awareness as to effective rabies control strategies	Animal health workers and community members
Education: Limited availability/accessibility of rabies educational material in poorly resourced countries	At-risk communities, through global networks
Advocacy and communication: Insufficient dialogue/information sharing among global rabies workers/leaders	Global networks, key opinion leaders and experts, governments
Social mobilization and community outreach: Insufficient rabies prevention and control efforts because rabies affects “neglected” communities	Local communities
Social mobilization and community outreach: Lack of priority given to rabies prevention and control at the central level	Local communities

First column extracted and second column adapted from Table 1 in Lembo et al. [8] where World Rabies Day was listed as one of the programmes to address gaps in rabies prevention

One of the main problems with preventing rabies at the most basic level is a lack of knowledge among people living in rabies-endemic areas –that they need to vaccinate their dogs to prevent the disease at source, that they should wash any wound and seek medical attention following animal bites, and that acting on this knowledge can save lives. Individuals and organisations working on this issue across the world are often isolated and an inclusive platform can help stakeholders in rabies control and prevention efforts to share resources and successes, and gain recognition and support.

As a neglected disease, rabies does not attract the support and resources needed to eliminate the disease. Health awareness days can encourage a social and political environment to support policy changes [9], thus helping to prioritise rabies and attract resources for prevention and control programmes. World Rabies Day was therefore conceived as a day of education and action allowing rabies prevention messages to be tailored and delivered to a range of different audiences, and to connect disparate groups working towards the same goal.

## How World Rabies Day works

GARC set up World Rabies Day in 2007 as a joint initiative with the US Centers for Disease Control and Prevention (CDC), with the co-sponsorship of the WHO and the OIE, and the support of the Pan-American Health Organization (PAHO) and the FAO. Except for the first year, it has been observed every year on September 28th, the anniversary of the death of Louis Pasteur, who developed the first successful rabies vaccine.

World Rabies Day was designed to be an open, global, inclusive platform to encourage stakeholders at all levels to take part in a unified day of action against rabies. The objectives of the first World Rabies Day campaign were to:

- Raise global awareness about rabies and how to stop the disease
- Educate people in rabies-endemic countries, especially children and health professionals
- Mobilise resources to support local rabies prevention programmes

GARC acts as a facilitator, supporter, promoter and aggregator of activities (termed World Rabies Day ‘events’) to amplify the commitment of the global rabies community, and to increase attention from the media and policy makers.

Given the limited resources available to GARC, both in terms of personnel and funds, a three-pronged approach was taken to develop the campaign:

1. Development of the World Rabies Day event platform and global toolkits to provide materials and resources for individual community members to take action.
2. Promotion and distribution of World Rabies Day messages by GARC and through key rabies stakeholders.
3. Year-specific activities actively managed by GARC to highlight specific issues.

### World Rabies Day platform and toolkits

#### An inclusive web-based platform

The fundamental resource provided by GARC to promote events is a web-based platform, in English, Spanish, Portuguese and French, where anyone can register a World Rabies Day event and add information and photos [10]. This generates an individual public page for each event, which can then be shared with others to increase participation, and also to attract attention from the broader rabies community, including potential collaborators and donors. The database of past event organisers allows GARC to distribute World Rabies Day updates and new resources directly to those most active in the field of rabies prevention.

In 2013, the World Rabies Day website was incorporated into the GARC website to more fully integrate the campaign into GARC’s broader remit of work. At this time, the method of recording events was improved.

#### Event branding

**Fig. 1:**
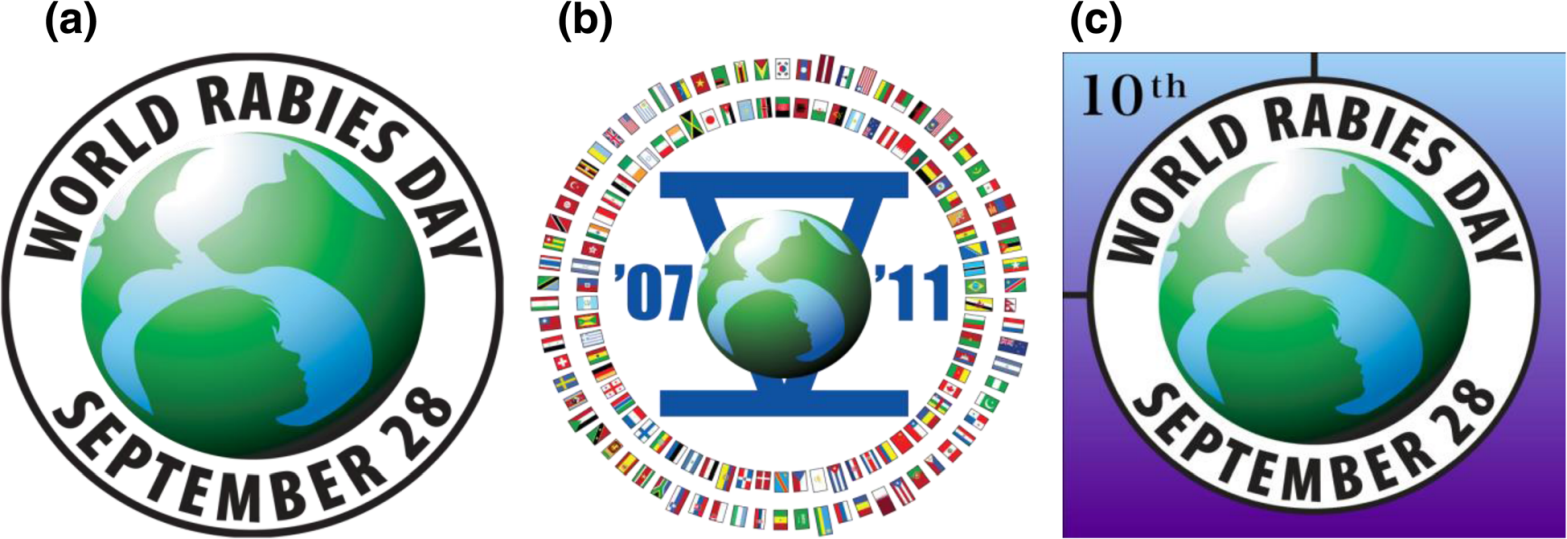
World Rabies Day logos. **a** the Forever logo; **b** logo created for the 5th World Rabies Day and; **c** logo created for the 10th World Rabies Day

World Rabies Day has its own distinctive logo, initially available in around 30 languages, and now downloadable in 51 languages from the GARC website. The World Rabies Day logo highlights the nature of the disease, a threat to domestic animals, human beings and wildlife. For the 5th (in 2011) and 10th (in 2016) World Rabies Days, special logos, still maintaining the characteristic central element, have been created (Fig. 1).

Each year the campaign unites under a common theme that symbolises a key message in rabies prevention. For example, the 2015 theme was ‘End Rabies Together’, to underline that intersectoral collaboration is needed to eliminate rabies and that everyone has a role to play in ending rabies deaths. The 2016 theme, ‘Rabies: Educate. Vaccinate. Eliminate.’ follows the key outcome of the Global Rabies Meeting held in December 2015, a strategic framework with the goal of eliminating human deaths due to canine-mediated rabies by 2030 [11].

Increasingly, social media has been incorporated into the World Rabies Day campaign to connect with new audiences. Hashtags such as #TogetherAgainstRabies, #WorldRabiesDay and #EndRabiesTogether have been utilised by GARC, its partners and the community across various social media platforms, particularly Facebook and Twitter. These tags enable people to be connected to others using the same hashtag, and allow GARC to collate a real time cross-section of what people are doing for and saying about World Rabies Day.

#### World Rabies Day toolkits

The GARC website provides several free downloadable toolkits to organisers that provide guidance around holding an event, ideas for events that can be adapted to different settings, key messages, event planning ideas and checklists. A Communications Toolkit guides organisers through how to involve the media and use social media for maximum impact. Background information on rabies, and the history and impact of World Rabies Day is also provided. A Policy Toolkit describes how to ask policy makers to create change and includes tools to support this process.

#### Educational resources

To respond to the lack of awareness about appropriate rabies prevention strategies, developing and increasing access to basic awareness and educational resources for different audiences has been another important part of World Rabies Day support. Children’s lessons, toolkits for medical and veterinary clinics, and awareness posters in different languages are among the materials created over the years.

Following feedback after the first World Rabies Day, the campaign created a central web-based database for rabies prevention materials from all over the world, categorised and searchable by geographic region, language, audience and resource type, providing a single port of call for rabies prevention resources available to download free of charge [12]. World Rabies Day has also been used as an opportunity to promote other educational initiatives, such as the GARC Education Platform, which currently offers the Rabies Educator Certificate and the Animal Handling and Vaccination Certificate [13].

### Promotion and distribution of World Rabies Day messages

Rabies is a problem that usually only hits the headlines when unusual or large outbreaks occur. Being a unified day of action, World Rabies Day provides a much needed media hook where more positive messaging about progress in rabies prevention and control can be promoted. It can also bring attention to the global situation in countries where people no longer have to live in fear of rabies.

GARC reaches out by email each year to its newsletter database, previous World Rabies Day event organisers, and partner networks. The campaign relies strongly on partner organisations to share information, promote their own activities and distribute key World Rabies Day messages across their networks, and make media announcements. Through these extensive networks and media opportunities, the reach of World Rabies Day is amplified dramatically.

The World Rabies Day team is increasingly engaged with social media. As well as using Facebook and Twitter to advertise World Rabies Day, encourage engagement in events and highlight achievements, the team also tests ways to use these media to reach new audiences. Again, partner organisations are encouraged to amplify messages through their own social media channels.

### Year-specific activities

To keep World Rabies Day fresh, and to appeal to different audiences, various GARC-driven activities have been carried out over the years. These have been made possible by specific funding from partner organizations and have included poster distribution, international webinars, event competitions and social media campaigns.

Strategies within these approaches have been modified according to experience, building on the most successful aspects of the year-specific activities to make the best use of very limited resources. One early strategy, to attract individual donations and fundraising efforts from developed countries to support efforts in canine rabies endemic regions, proved to be too time-consuming for the gains achieved, and efforts were re-focussed on the need to increase awareness to save lives and the wider advocacy that World Rabies Day could support.

## What World Rabies Day has achieved so far

From the first World Rabies Day in 2007, the extensive professional networks of GARC, US CDC and other major organisations involved in rabies control, including the WHO, OIE, FAO and PAHO, have been utilised. The campaign has reached out to medical and veterinary associations (and their student chapters), animal welfare organisations and vaccine manufacturers.

### Registered events across the world

The World Rabies Day themes chosen each year are designed to be widely applicable. Rabies stakeholders from many sectors and levels, including national and local governments, international and community-based non-governmental organisations (NGOs), colleges and schools, medical and veterinary professionals and scientists have registered events. The nature of the events held has varied considerably, from rabies awareness sessions at local schools, to local distribution of rabies prevention information, mass dog vaccination campaigns, professional development seminars and national media events [14].

The initial target of the 2007 World Rabies Day was to engage 55,000 people in rabies prevention. At the time, this was the estimated number of people dying of rabies each year. For this first year, a total of 139 events in 48 different countries were registered. Feedback from a follow-up survey sent to event organisers suggested that an estimated 400,000 people in 74 countries participated in some way in World Rabies Day 2007 [15].

**Fig. 2:**
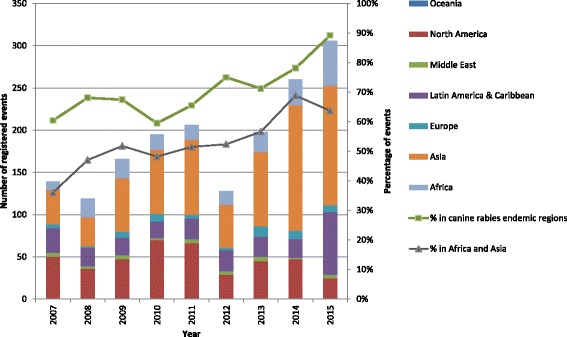
World Rabies Day Events 2007–2015

In total, from 2007 to 2015, a total of 1,717 events from 116 countries have been registered on the GARC World Rabies Day platform with a general increase over time, but with drops in 2008 and 2012 (Fig. 2). Between 2007 and 2015, the percentage of events held in canine rabies endemic regions rose from 60 to 89 % of events, and those held in Africa and Asia from 36 % to over 60 %. The 2012 dip follows in part an organisational decision to move away from active promotion in the US to focus on endemic country outreach.

Whilst the number of registered events gives an indication of the success of the campaign, it does not reflect the true picture of outreach to communities. Many organisers register a set of events across a network, organisation or country as one event, which leads to under-representation of activities. For example, a single event registered in 2015 for Bhutan incorporated the airing of a documentary video about rabies through Bhutan Broadcasting Service, text messages about rabies across mobile networks, a live TV panel discussion on rabies, and radio talks on rabies in three languages, along with print media efforts and free dog and cat vaccination clinics, over a 3-day period.

GARC often finds out about unregistered events after they have occurred, particularly during conference presentations about rabies, where World Rabies Day events are mentioned and the logo used. This suggests that the campaign outreach may be wider than recorded, although it is not possible to say how common unregistered events and activities are. Finally, it is possible that some over-reporting may occur if registered events do not ultimately take place.

Governments of rabies-endemic countries have increasingly used World Rabies Day to highlight their efforts and announce major rabies-related initiatives, both to reach in-country audiences as well as the international community. In 2007, Peru celebrated World Rabies Day by issuing a commemorative stamp, and the Nigerian Minister of Health did a national television broadcast on rabies. In 2010, the Botswana Department of Veterinary Services and other national ministry and international partners led an event that involved a march of over 100 people from the Ministry of Agriculture to the Gaborone Secondary School, where an all-day public information session was held.

Every year, the Philippines government announces rabies-free zones on World Rabies Day. In 2015, a memo from the Philippines National Government was issued to all provincial, city and municipal governments to encourage them to hold events to observe World Rabies Day. In the same year, the Mexican Health Ministry issued a commemorative lottery ticket for World Rabies Day, and Kenya used World Rabies Day as the springboard to launch its national rabies elimination strategy [16].

World Rabies Day also helps to build opportunities for more comprehensive rabies prevention initiatives. In 2015, what began as a request by the US Naval Ship Comfort for educational resources to distribute in Haiti during World Rabies Day developed into a full scale workshop on Haiti’s national rabies strategy with government representatives and partners including Continuing Promise 2015, US Naval Ship Comfort, US CDC, Christian Veterinary Mission, PAHO, Humane Society International and the International Fund for Animal Welfare.

### Outreach through partnerships

For World Rabies Day, GARC secured the involvement of the major intergovernmental agencies involved in rabies control. The WHO, OIE and FAO and other organisations send World Rabies Day information out to their networks. The Regional offices of the WHO, OIE, FAO and WHO/OIE rabies Collaborating Centres have also actively contributed to the outreach. The three international health agencies and the US CDC also have dedicated World Rabies Day sections on their websites and have issued media releases around the day. Notably in 2013, the FAO, WHO and OIE jointly released a statement on World Rabies Day where they first called for the global elimination of canine-mediated rabies [17].

Many other organisations have distributed World Rabies Day messages to their networks, including: US CDC, Fondation Merieux, World Animal Protection (formerly the World Society for the Protection of Animals -WSPA), One Health Initiative, American Veterinary Medical Association, World Veterinary Association (WVA), International Veterinary Students’ Association (IVSA), International Federation of Medical Students’ Associations (IFMSA), and World Small Animal Veterinary Association (WSAVA). Corporate partners, such as Sanofi Pasteur, Novartis, Merial and MSD Animal Health, have also played a significant role in distributing messages through international headquarters and national offices. In addition, campaign updates are sent out to the GARC database of almost 3000 subscribers who are involved in rabies prevention and control.

The World Rabies Day toolkits encourage event organisers to partner with their local media to generate visibility for their activities. World Rabies Day has also attracted media attention from globally respected outlets including The Lancet [18], the Telegraph newspaper [19], the Journal of the American Veterinary Medical Association [20], the Veterinary Record [21] and the Independent newspaper [22].

### Year-specific activities

A variety of different initiatives have been carried out over the years to keep World Rabies Day appealing and to attract different audiences. One of World Rabies Day’s first initiatives was the professional development of a complete package of lessons for children, ‘All about Rabies’, with customisable content. This package was adapted and used by organisations in Ghana, Kenya and Egypt, with GARC’s support. GARC held international webinars for World Rabies Day from 2010–2012. Covering advances in rabies control and research, these webinars reached professionals working in rabies control and other interested parties across 80 countries over 3 years.

In 2011, GARC received funding to develop educational outreach in Africa, and a partnership was formed with the FAO to distribute posters in different languages through their African offices. Basic materials are often lacking in many countries, and although posters and other resources can be freely downloaded, it is not always easy to access the internet, and funds are not always available to print them in the quantities required to reach their target population. Between 2012 and 2013, a total of 17,915 posters and 31,060 leaflets were sent to organisations in 12 African countries.

World Rabies Day has always promoted the One Health approach, and has actively encouraged collaboration between sectors. For example, in 2014, GARC, in collaboration with the IFMSA and IVSA, held a Global One Health Challenge, a competition for veterinary and medical students working together, with the winning team attending a conference hosted by the World Medical Association and the WVA. There were 29 entries that reached over 1,600,000 people with rabies prevention messages and resulted in the vaccination of over 12,000 animals.

More recent World Rabies Day initiatives were built around social media, such as GARC’s 2014 *Me and My Dog* campaign to encourage people to keep their dogs vaccinated, by sharing photos of themselves and their dogs on our platforms [23]. This initiative actively engaged 123,500 people, was published on the timelines of over 6 million people and trended nationally in India (where the campaign was focused). It also generated a 700 % increase in Facebook followers to the GARC account and an ongoing ability to reach these people in future.

In 2015 World Rabies Day encouraged its social media followers to sign up to a ‘Thunderclap’, which provided the right to post a World Rabies Day message to their timeline at the same time as all the other participants and thereby amplify the impact of the message, which reached 246,086 social media accounts [24].

### International adoption of World Rabies Day

Generating impact beyond that recorded by the events held, World Rabies Day is now viewed as an integral part of rabies elimination programmes, a day to make major rabies-related announcements and to increase rabies awareness globally.

In 2007, regional collaboration in the Americas saw PAHO staff participating in national events in Argentina, Bolivia, Brazil, Chile, Colombia, Costa Rica, Dominican Republic, Ecuador, El Salvador, Guatemala, Haiti, Honduras, Mexico, Nicaragua, Paraguay, Peru and Venezuela [25]. Specific activities included:

- Brazil: National media campaign with the Ministries of Health and Agriculture, and Rabies Surveillance Forum
- Chile: The PAHO representative and the Undersecretary of Public Health held a press conference
- Costa Rica: Media and awareness campaign held, targeting school children, organised by WSPA and PAHO
- Haiti: Ministers of Public Health and Agriculture held a press conference with the Haiti PAHO Representative

In 2009 the European Commission launched their annual Veterinary Week on World Rabies Day, highlighting its importance as the embodiment of One Health and encouraging the collaborative participation of veterinary and medical organisations throughout Europe. In Asia, the Association of Southeast Asian Nations (ASEAN) has integrated World Rabies Day into the ASEAN Rabies Elimination Strategy [26]. ASEAN also released a call to action on World Rabies Day 2014 for the regional rabies elimination date of 2020 [27].

In Africa, the Africa Rabies Expert Bureau, a network of rabies experts from Francophone African countries, added World Rabies Day to its resolutions at its March 2009 meeting – *To increase rabies awareness, by actively participating in the World Rabies Day* [28]. This network later became part of the Pan-African Rabies Control Network (PARACON). At the first PARACON meeting in 2015, recommendations from the 33 African countries that attended included one to: *Consider World Rabies Day (September 28th) an opportunity for rabies advocacy on all levels concerned - community, provincial, national, regional and beyond* [29].

In the Americas, PAHO, in collaboration with GARC, provided awards for the most outstanding World Rabies Day initiatives in Latin America and the Caribbean for the first four years of World Rabies Day. In 2014, the Pan-American World Rabies Day Initiative was launched, to increase awareness about rabies prevention among professionals and relevant government departments in the Americas. This is a collaboration between regional veterinary medical associations, NGOs and PAHO.

At the global level, in 2011 the OIE’s Global Conference for Rabies Control recommendations included one encouraging OIE Member Countries to support awareness campaigns on rabies (e.g. participate in the World Rabies Day initiative) [30]. The tripartite joint call for canine rabies elimination on World Rabies Day in 2013 was a significant political step for global rabies control [17] and in December, 2015, a new global framework to eliminate rabies (WHO, OIE, FAO, GARC), listed World Rabies Day as a key intervention under the socio-cultural pillar of the five-pillar framework [11]. The endorsement of World Rabies Day by WHO has led to it being listed as an International Day observed by the United Nations [31].

## Reflections

The success of the first World Rabies Day showed that it was embraced by the global rabies community. The event’s success benefitted hugely from the large professional networks of the initial organisers at GARC and the CDC, all experienced rabies scientists. The co-sponsorship of international agencies ensured take-up at the national, regional and international levels. By developing shareable tools and highlighting that the day belonged to the community, rabies stakeholders took ownership of the concept, helped by the broad scope of the World Rabies Day objectives, and developed ideas and initiatives to meet their needs.

World Rabies Day has helped to fill the gaps mentioned in Table 1, through a combination of a virtual platform and resources to support local efforts around the world and strategic partnerships to use existing networks to amplify the messages. It has registered thousands of events, reaching millions of people every year, though exact outreach and impact are very hard to judge, especially given the huge diversity in events and their scope. For a few years participant feedback was sought, but this adds even greater burdens to event organisers and the data collected could not be verified by GARC. Despite these issues, World Rabies Day clearly promotes and supports unique partnerships, provides a forum for organisers to connect and see their work as part of a global effort, and shows donors and politicians the strength and commitment of the rabies prevention community.

There have been a few major challenges that have had to be addressed over the years, one of the biggest being the extremely limited resources available, both in terms of personnel and funds. GARC tries to address this by seeking funding partnerships for specific initiatives, and providing a central point of contact to provide at least basic support where this is not possible. The GARC platform also helps to increase visibility and fosters link-building at all levels, whether between international organisations or grassroots advocates. There is also the ongoing challenge of participant fatigue, especially as the day matures. This not only affects community take up of activities, but also the donor community.

Due to time and resource constraints, World Rabies Day moved away from one of its initial aims- to encourage and support community-based fundraising activities. Whilst a few such activities still occur, GARC has focused more on the value of World Rabies Day in raising awareness and advocacy for improved rabies control efforts coordinated by governments. This is reflected in the drop off of engagement in 2012 as GARC shifted from North America based events to better align activities with its mission to raise awareness and promote education in rabies-endemic countries. GARC further strengthened the education component by bringing World Rabies Day under GARC’s broader work towards rabies prevention.

A global day with planned local impact faces the challenges of bridging many different languages and diverse cultures. Where possible, translations of logos and modifiable resources have been offered to the rabies community, and local volunteers and organisations have been utilised to provide support.

Besides language and culture, the move towards rabies-endemic countries has also raised issues around adequate internet access. GARC’s World Rabies Day support is primarily online, and many of the key target audiences do not have access to the internet. There are also questions about the literacy of some communities at risk. Wherever possible these challenges have been addressed by providing resources to local organisations that work in these areas and have internet access and the ability to suitably translate and adapt messages for their settings.

## Conclusions

The global rabies situation has changed substantially over the last ten years, and GARC’s strategy, including World Rabies Day, has adapted over time both to help create and to adapt to changes in the landscape. Building on its most successful experiences, World Rabies Day will continue to maintain its supporting function for individuals and organisations in rabies-endemic countries with its event platform and resources, to provide rabies prevention information to the community through institutions such as schools, local government and NGOs. GARC will continue to develop new partnerships for World Rabies Day and explore new ways of reaching its audiences. For example, 2016 will introduce regional awards for rabies prevention, customisable posters and Facebook event registration in response to feedback from event organisers.

World Rabies Day has recently been endorsed by governments from all over the world, and is now an integral part of the strategy towards global rabies elimination. It provides a unified day of action, principally in endemic countries, to refresh knowledge, remind people to vaccinate (and revaccinate) their animals, to highlight local rabies prevention initiatives and to call for increased control efforts. The news and media attention that World Rabies Day generates feed into longer term campaigns such as the roll out of the recent global framework and End Rabies Now, a new GARC-coordinated multi-partner year-round campaign, building political pressure to work towards zero human deaths from canine-mediated rabies by 2030.

World Rabies Day has been fully integrated into GARC’s programmatic activities in rabies-endemic countries, where it is a tool to help build in-country capacity to encourage and support governments to make rabies prevention awareness a priority within rabies elimination programmes, to help them to meet the 2030 elimination date. It can be used as a day to celebrate regional progress and encourage competition among countries. Of course, there are likely to be ongoing challenges, such as limited resources, competing health days and other government priorities, but World Rabies Day has earned its position within the strategy towards global rabies elimination and will continue to be relevant to stakeholders and audiences at all levels in rabies-endemic countries.

## Abbreviations

ASEAN: Association of Southeast Asian Nations
CDC: US Centers for Disease Control and Prevention
FAO: United Nations Food and Agriculture Organization
GARC: Global Alliance for Rabies Control
IFMSA: International Federation of Medical Students’ Associations
IVSA: International Veterinary Students’ Association
NGOs: Non-governmental organisations
OIE: World Organisation for Animal Health
PAHO: Pan-American Heath Organization
PARACON: Pan-African Rabies Control Network
PEP: Post-exposure prophylaxis
WHO: World Health Organization
WSAVA: World Small Animal Veterinary Association
WSPA: World Society for the Protection of Animals
WVA: World Veterinary Association
